# DNA N6-Methyladenine Modification in Eukaryotic Genome

**DOI:** 10.3389/fgene.2022.914404

**Published:** 2022-06-24

**Authors:** Hao Li, Ning Zhang, Yuechen Wang, Siyuan Xia, Yating Zhu, Chen Xing, Xuefeng Tian, Yinan Du

**Affiliations:** ^1^ School of Basic Medical Sciences, Anhui Medical University, Hefei, China; ^2^ First School of Clinical Medicine, Anhui Medical University, Hefei, China; ^3^ First Affiliated Hospital of Anhui Medical University, Hefei, China; ^4^ Second School of Clinical Medicine, Anhui Medical University, Hefei, China

**Keywords:** methylation, DNA modification, N6-methyladenine, eukaryotic genome, epigenetics

## Abstract

DNA methylation is treated as an important epigenetic mark in various biological activities. In the past, a large number of articles focused on 5 mC while lacking attention to N6-methyladenine (6 mA). The presence of 6 mA modification was previously discovered only in prokaryotes. Recently, with the development of detection technologies, 6 mA has been found in several eukaryotes, including protozoans, metazoans, plants, and fungi. The importance of 6 mA in prokaryotes and single-celled eukaryotes has been widely accepted. However, due to the incredibly low density of 6 mA and restrictions on detection technologies, the prevalence of 6 mA and its role in biological processes in eukaryotic organisms are highly debated. In this review, we first summarize the advantages and disadvantages of 6 mA detection methods. Then, we conclude existing reports on the prevalence of 6 mA in eukaryotic organisms. Next, we highlight possible methyltransferases, demethylases, and the recognition proteins of 6 mA. In addition, we summarize the functions of 6 mA in eukaryotes. Last but not least, we summarize our point of view and put forward the problems that need further research.

## Introduction

DNA methylation is one of the most important epigenetic modifications, and is involved in various biological progresses. Previously, research mainly focused on 5-methylcytosine (5 mC). 5 mC is the earliest and best-studied DNA methylation modification in eukaryotes and for most eukaryotes, the abundance of 5 mC in CpGs is over 50% (Chen et al., 2018; Schmitz et al., 2019). In vertebrates, the detected 5 mC level of CpGs is over 70% (Feng et al., 2010). 5 mC is widely involved in transcription suppression, transposon suppression, genomic imprinting, X chromosome inactivation, and epigenetic memory (Bird, 2002; Chen et al., 2016; Wu and Zhang, 2017). Compared with 5 mC N6-methyladenine (6 mA) was considered to exist only in prokaryotes for a long time and has recently been discovered in some eukaryotes with a low prevalence. In prokaryotes, 6 mA plays an important role in distinguishing host DNA from exogenous DNA (Razin, 1984) and controls many biological functions, such as DNA replication, transcription, mismatch repair, chromosome replication, nucleoid organization and segregation, phase variation, bacterial conjugation, and bacterial virulence (Reisenauer et al., 1999; Wion and Casadesús, 2006; Vasu and Nagaraja, 2013). With the development of detection techniques, 6 mA was reported to be present in an increasing number of eukaryotes, including *Chlamydomonas* (Fu et al., 2015), *C. elegans* (Greer et al., 2015; Ma et al., 2019), *Tetrahymena* (Wang et al., 2017), ciliates (Beh et al., 2019), fungi(Mondo et al., 2017), *Arabidopsis Thaliana* (Liang et al., 2018), rice(Zhou et al., 2018), *Drosophila* (Zhang et al., 2015; Shah et al., 2019), mice (Yao et al., 2017; Kweon et al., 2019), rats (Kigar et al., 2017), zebrafish (Liu et al., 2016b), and humans(Wu et al., 2016; Xiao et al., 2018; Hao et al., 2020).

It has been demonstrated that 6 mA plays an increasingly important role in eukaryotes. Recently, studies of 6 mA methylation have gradually advanced, and a growing number of methyltransferases have been discovered. However, enzymes involved in 6 mA demethylation in eukaryotes are still scarce, and the proteins identifying 6 mA sites remain to be explored. In this review, we first discuss the advantages and disadvantages of 6 mA detection technologies and the prevalence of 6 mA in eukaryotic organisms. Then, we highlight the possible methyltransferases, demethylases, and proteins recognizing 6 mA. Finally, we summarize the functions of 6 mA and put forward the problems that need further research.

## Detection of 6 mA

Over the past few decades, multiple methods have been developed to detect 5 mC at a single-gene level or whole-genome level based on sodium bisulfite transformation, chromatography, methylation sensitive restriction enzymes, 5 mC methyl-binding proteins or antibodies to 5 mC, as well as rapid and inexpensive biosensors for detection (Lv et al., 2021; Martisova et al., 2021). The detection methods of 6 mA and 5 mC have many similar principles. However, due to the low abundance of 6 mA and possible bacterial contamination, the sensitivity and reliability of detection technologies are challenged. Here, we discuss experimental tools and bioinformatics tools for 6 mA detection and their advantages, disadvantages, and limitations (Table 1).

**TABLE 1:** Advantages and disadvantages of 6 mA detection methods.

Detection methods	Sensitivity	Specificity	Detecting at single-base resolution	Implement ability	Weaknesses
6 mA-IPseq	relatively low	low	no	relatively low cost, easy to conduct	interferences of m1A, m6A, and enrichment of unmethylated DNA fragments
6 mA-REseq	relatively low	high	yes	relatively low cost, easy to conduct	limitation of specific restriction sites
HPLC-MS/MS	high	high	no	relatively complex operation, a requirement for instrument	possible bacterial contamination of enzymes
SMRT	high	relatively low	yes	incredibly costly	interferences of 1 and 6 mA, high false positive rate
Deep leaning	relatively high	relatively high	yes	low cost, save time	low confidence, limitations of the model derived from experimental data

### 6 mA-IPseq

**FIGURE 1:**
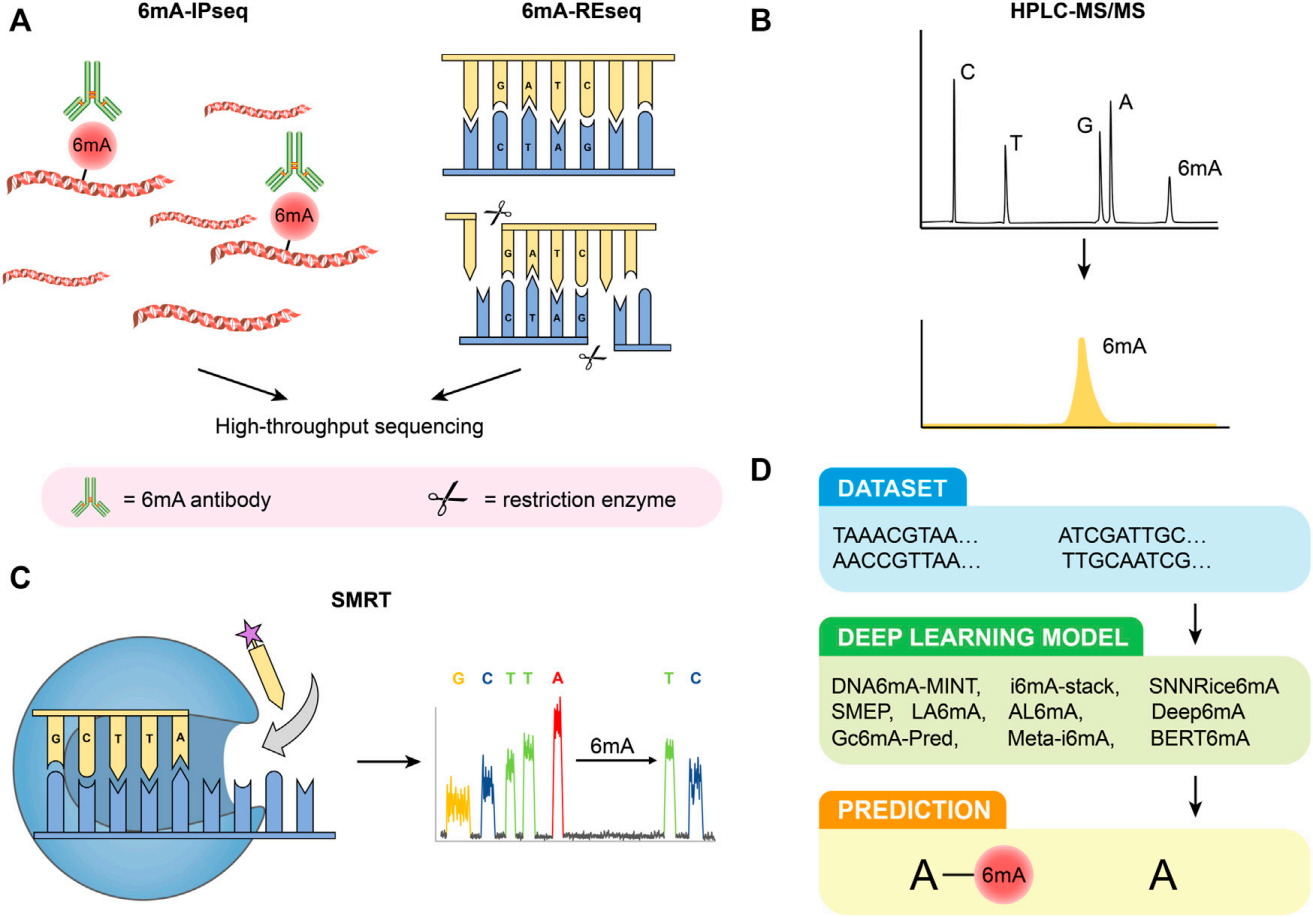
Detection methods of 6 mA. **(A)** 6 mA-IPseq and 6 mA-REseq. **(B)** HPLC-MS/MS. **(C)** SMRT. **(D)** Deep learning predictive model.

6 mA-immunoprecipitation sequencing (6 mA-IPseq) is a common method of methylation detection. It enriches methylated genomic fragments using a specific 6 mA antibody and then identifies DNA motifs by sequencing (Figure 1A) (Fu et al., 2015). The cost of 6 mA-IPseq is relatively low, however, the inability of 6 mA antibodies to precisely locate methylation sites limits the application of this method (Jeong et al., 2016). Recently, investigators reported the preference of 6 mA antibody to unmodified adenine, which indicated the possible false positive results caused by enrichment of unmethylated DNA (Douvlataniotis et al., 2020). In addition, N6-methyladenosine (m6A) or m1A in RNA also disturbs the test (Douvlataniotis et al., 2020). Furthermore, during cell culture, bacterial DNA containing 6 mA may be incorporated into samples DNA (Schiffers et al., 2017). Therefore, 6 mA-IPseq requires high-quality DNA samples without bacterial contamination.

### 6 mA-REseq

Restriction enzyme-based 6 mA sequencing (6 mA-REseq) relies on a collection of restriction enzymes that digest DNA motifs without specific methylation (Figure 1A). Genomic DNA treated with restriction enzymes is fragmented by sonication, end-repaired, and then ligated to DNA adapters. After PCR amplification, the DNA library can be prepared for high throughput sequencing. The unmethylated sequence motifs are enriched at the end of the sequencing reads while methylated motifs are present in the inner part of the reads. The ratio of internal motifs to terminal motifs reveals the relative methylation to unmethylation ratio (Fu et al., 2015). However, the application of 6 mA-REseq is limited to specific restriction sites, and incomplete digestion caused by other reasons may also lead to false positive results (Laird, 2010; Shanmuganathan et al., 2013).

### HPLC-MS/MS

High-performance liquid chromatography coupled with tandem mass spectrometry (HPLC-MS/MS) is a highly sensitive and specific method for 6 mA detection. Before being analyzed by HPLC-MS/MS, purified DNA samples are first digested by commercial enzymes. Thereafter the digested DNA can be effectively separated in the chromatographic separation system due to the different physical and chemical properties of each component. Next, they are ionized by atmospheric pressure ionization (API) techniques and then entered into the mass spectrometer, identified by MS/MS based on mass-to-charge ratio (m/z) (Vogeser and Seger, 2008; Liu and Wang, 2021) (Figure 1B). HPLC-MS/MS can accurately quantify the signal of each nucleoside even if the samples are contaminated by RNA(Song et al., 2005). However, the result of HPLC-MS/MS can be easily disturbed by bacterial contamination in samples and commercial enzymes (Schiffers et al., 2017; Koh et al., 2018; O'Brown et al., 2019). As a result, strict aseptic conditions and appropriate experimental control are necessary to ensure the accuracy and validity of the results.

### SMRT

Single-molecule real-time sequencing (SMRT) is based on DNA polymerases and fluorescence-labeled deoxyribonucleoside triphosphates (Figure 1C) (Morgan et al., 2009; Flusberg et al., 2010). In zero-mode waveguides, different fluorescently labeled deoxyribonucleoside triphosphates (dNTPs) are incorporated into the DNA chain by DNA polymerase. The type of dNTPs is determined by the type of fluorescence, and base modifications of DNA can be directly revealed by changes in inter-pulse duration (IPD) values, which means the interval between fluorescence pulses (Flusberg et al., 2010). The development of SMRT provides a more powerful tool for the direct detection of modified nucleotides in DNA. However, the high false positive rate (FPR) of SMRT, especially when the abundance of 6 mA/A is low, has attracted the attention of researchers (Zhu et al., 2018; O'Brown et al., 2019; Douvlataniotis et al., 2020). SMRT cannot distinguish between 6 and 1 mA, and modifications of flanking cytosine may also cause interference (Schadt et al., 2013; Douvlataniotis et al., 2020). The high FPR of SMRT is dependent on the 6 mA rate over the adenines (6 mA/A) in the genome and the sequencing depth and coverage (average of IPD values for each strand of the genome reference). Considering the low level of 6 mA/A in eukaryotes, deep coverage is indispensable to attain a low FPR (Zhu et al., 2018). In addition, whole genome-amplified DNA (WGA DNA, unmethylated DNA) is also recommended as a control to reduce FPR (Yang et al., 2020). SMRT is also suggested to be used in combination with other detection methods.

### Deep Learning Predictive Model

Compared with traditional laboratory experiments, bioinformatics tools have significant advantages in terms of price and time cost (Figure 1D). At present, there are many deep learning models used for predicting 6 mA, such as DNA6mA-MINT (Rehman and Chong, 2020), i6mA-stack (Khanal et al., 2021), SNNRice6mA (Yu and Dai, 2019), SMEP (Wang et al., 2021), Deep6mA (Li et al., 2021b), LA6mA, AL6mA (Zhang et al., 2021), GC6mA-Pred (Cai et al., 2022), Meta-i6mA (Hasan et al., 2021), and BERT6mA (Tsukiyama et al., 2022). Based on neural networks, Yu and Dai. (2019). proposed a new method called SNNRice6mA to identify 6 mA sites in rice DNA, which showed over 90% sensitivity, specificity, and accuracy. However, the accuracy of SNNRice6mA for cross-species studies decreased significantly, from 93% and 92% in two types of rice to 61.81% in *Mus musculus*. Other algorithms also have their characteristics. For example, Deep6mA presents an accuracy of more than 90% in predicting plants such as *Arabidopsis* (Li et al., 2021b). LA6mA and AL6mA capture location information from DNA sequences through a self-attention mechanism (Zhang et al., 2021). GC6mA-Pred mainly identifies 6 mA sites in the rice genome and outperforms several prediction models, including DNA6MA-MINT, on independent datasets (Cai et al., 2022). These methods present many advantages; however, there are also some problems. BETR6mA was less effective in species with small sample sizes and required pretraining and fine-tuning(Tsukiyama et al., 2022). Although deep learning models present high accuracy and sensitivity in particular species, they are doubtful when they are extrapolated to other species. In the future, with the continuous optimization of deep learning models, they will play an important role in predicting 6 mA sites.

### Prevalence of 6 mA

Chemical modification of nucleotide bases in DNA conveys added information to the genetic code. As the most common chemical modification, 5 mC is widely present in higher eukaryotes, such as plants, protozoans, metazoans, and some fungi (Schmitz et al., 2019). In most species of plants, such as tomatoes and oranges, 5 mC is tissue-specific and varies during the growth of plants (Chachar et al., 2021). In vertebrates, the genomes are extensively methylated, where the detected 5 mC of CpGs is more than 70% (Feng et al., 2010). In the mammalian genome, 5 mC primarily occurs within the CpG dinucleotide context, and 60%–80% of CpGs are methylated (Smith and Meissner, 2013; Luo et al., 2018a). However, it was almost undetectable in *Drosophila* and *C. elegans* Chen et al., 2018). In fungi, Bewick et al. (2019). analyzed the prevalence of 5 mC in 40 fungal species and discovered that the level of 5 mC in *Basidiomycota* was the highest whether in a genomic location or sequence context. Whereas 5mC was nonexistent in common fungi such as *Saccharomyces cerevisiae* (a species of yeast) *and Aspergillus nidulans.* In addition, fungi were reported to lack canonical gene-body methylation, which meant 5 mC was not evenly distributed.

**FIGURE 2:**
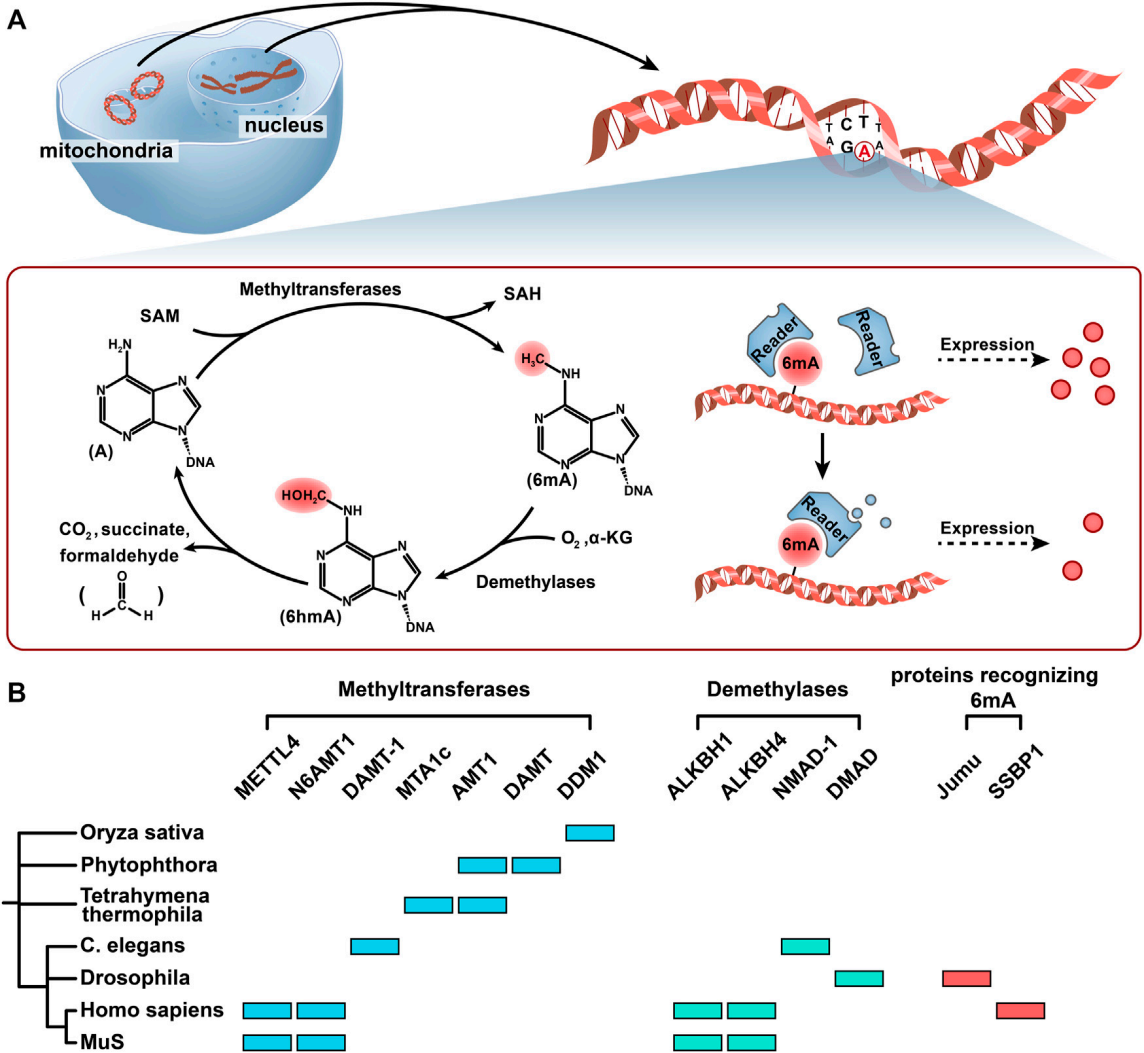
Methylation and demethylation of 6 mA. **(A)** The methyl group on SAM was added to the sixth position of the adenine ring primarily with the help of methyltransferases. Under the catalysis of demethylase ALKBH1, 6 mA is oxidized to the 6 hmA intermediate by Fe^2+^, O_2_, and α-KG, and then 6 hmA spontaneously degrades to adenine and generates formaldehyde without the catalysis of demethylase. Readers (proteins recognizing 6 mA) may recognize the 6 mA modification and manipulate the fate of 6 mA-modified genes in different cellular contexts. **(B)** Seven kinds of 6 mA methyltransferases, four kinds of 6 mA demethylases, and two kinds of proteins recognizing 6 mA in 7 different organisms are shown in a simplified phylogenetic tree. Color codes represent the methyltransferases, demethyltransferases, and proteins recognizing 6 mA in the corresponding organism and proteins.

Compared to 5 mC, 6 mA was detected extensively in prokaryotes (Figure 2A). In eukaryotes, the existence of 6 mA is controversial. Recently, some research has shown the existence of 6 mA in eukaryotes, including protozoans, metazoans, plants, and fungi. In different biological genomes, the abundance of 6 mA is quite different. In 2020, Lizarraga et al. reported that 6 mA accounted for 2.5% of the total adenine in the parasite *Trichomonas vaginalis*. They also demonstrated that 6 mA was mainly located in intergenic regions (94% of 6 mA-IPseq peaks). Among the 6 mA peaks located in genes (6%), most were distributed between the coding region (48%) and the transcription termination sites (TTSs; 43%), with only 9% found in the TSSs (Lizarraga et al., 2020). In *Drosophila*, the 6 mA level peaked (∼0.07%, 6 mA/A) at the 0.75-h embryonic stage and then decreased to a low level (∼0.001%, 6 mA/A) at 4–16 h embryonic stages (Zhang et al., 2015). However, in a more recent publication, it was demonstrated that the real level of 6 mA/A in total genomic DNA (gDNA) was 2 parts per million (p.p.m.) (CI, 1–10 p.p.m.) suggesting that previous measurements could be affected by bacterial contamination (Kong et al., 2022). In *Bdelloid rotifer*, 6 mA existed on 17,886 adenines (0.0236% of total adenines) (Rodriguez et al., 2022). In *C. elegans,* 6 mA accounted for 0.7% of the total adenine in the genome by SMRT sequencing (equivalent to 0.3% adenine methylated), which was further confirmed by UHPLC-MS/MS (Greer et al., 2015). In *Tetrahymena*, 6 mA was highly enriched in the NATN motif at linkers and transcription start sites (TSSs) (Wang et al., 2017; Luo. et al., 2018b). However, 6mASCOPE showed that the 6 mA/A level of VATN sites was 2–3 times higher than that of NATN sites (Kong et al., 2022). Compared to protozoans and metazoans, 6 mA has been less researched in plants and fungi. In 2018, Zhang et al. (2018). adopted multiple methods, including LC-MC/MC, 6 mA-IPseq, and 6 mA-REseq, and revealed that the 6 mA level ranged from 0.15% to 0.55% in rice seedlings. In addition, they also found that 6 mA was widely distributed in the *Japonica* and *Indica* genomes and enriched in promoters and exons. In *Arabidopsis*, Liang et al. (2020). reported that the level of 6 mA was up to 0.048% (6 mA/A) by LC-MS/MS. Kong et al. (2022). quantified the 6 mA/A level in 21-day-old Arabidopsis seedlings (approximately 2,500 p.p.m. 6 mA/A by LC-MS/MS). However, using 6 mASCOPE, they found that Arabidopsis only contributed to 4.21% of the total 6 mA events (3 p.p.m.; CI, 1–10 p.p.m.) and others were probably from four soil bacteria (Proteobacteria, Actinobacteria, Bacteroidetes, and Firmicutes). In sea buckthorn (*Hippophae rhamnoides Linn.*), the level of 6 mA was 0.016% by using nanopore sequencing at single-base resolution (Zhang et al., 2022a). Fu et al. 2015. reported that 6 mA-marked genes accounted for 84% of all genes in *Chlamydomonas*, and 6 mA was enriched in TSS. The existence of 6 mA has also been reported in Fig (*Ficus Carica L.*) (Usai et al., 2021). In fungi, Mondo et al. researched almost all the phyla of early-diverging fungi (EDF) and the Dikarya phyla and found that the abundance of 6 mA in EDF accounted for 2.8% of all adenines by SMRT, whereas the 6 mA level in the Dikarya could be a false positive by 6 mA-IPseq (Mondo et al., 2017). In EDF, 6 mA was symmetrically methylated, mainly present in the ApT context, and had a high density in methylated adenine clusters (MACs), whereas none of these were found in the Dikarya. In contrast to other EDFs, the 6 mA level in the arbuscular mycorrhizal fungi (AMF; Glomeromycotina) genome was 0.12%–0.17%, which was lower than that in other EDFs and similar to that in Dikarya and other eukaryotes (Chaturvedi et al., 2021).

The discovery of 6 mA modification in mammalian DNA has become a major focus of scientists. During embryogenesis in pigs, the level of 6 mA undergoes dynamic changes (Liu et al., 2016b). 6 mA gradually accumulated, reaching a maximum of ∼0.17%, and then decreased to 0.05%. They also reported a low abundance of 6 mA in adult pig tissues. Similarly, 6 mA was detected in mouse embryonic stem cells (ESCs). In the H2A. X deposition regions, the 6 mA level presented an abundance of ∼25–30 p.p.m. (Wu et al., 2016). In contrast, the level of 6 mA showed a linear increase in the embryonic states of mice and zebrafish (Fernandes et al., 2021). However, another group of researchers could not detect 6 mA in mouse ESCs or other tissues, which aroused extensive discussion (Schiffers et al., 2017). 6 mA existed in all of the brain regions and significantly increased up to 25.5 p.p.m. in the PFC upon stress. In 2018, Xiao et al. (2018). reported that the density of 6 mA was 0.051% in the human genome by SMRT sequencing, and the LC-MS/MS result was ∼0.056%. It was also reported that the 6 mA reached a level of 1,000 p.p.m. in glioblastoma stem cells and primary glioblastoma (Xie et al., 2018). However, the 6 mA level was found to be only 2 p.p.m. (CI, 1–16 p.p.m.) and 3 p.p.m. (CI, 1–13 p.p.m.) by 6mASCOPE in the two glioblastoma species, suggesting that the 6 mA level in human cells might be overestimated (Kong et al., 2022). Zhang et al. 2022b. identified 2,373 unstable methylated genes containing 6 mA and 5 mC modifications after comparing the methylated genes in HCC (hepatocellular carcinoma) and adjacent liver tissues. These results suggest that 6 mA may play an important role in human disease. Recently, 6 mA was found to be enriched in mitochondrial DNA (mtDNA) in humans. However, the distribution characteristics of 6 mA in mtDNA are debated. Koh et al. (2018). reported that 6 mA was enriched in the heavy strand of mtDNA and arranged throughout the entire mtDNA with no bias toward any specific region. In contrast, Hao et al. (2020). discovered that 6 mA was distributed in the promoter region and enriched in the ND2, COI, and ND4–ND6 regions.

The discussion above has demonstrated the existence of 6 mA in plants, protozoans, metazoans, and some fungi; however, some investigators believe that the current evidence is still insufficient due to bacterial contamination, interfering factors, and high FPR (Liu et al., 2017; O'Brown et al., 2019; Douvlataniotis et al., 2020). They believe that 6 mA should be considered a methylation modification only in basal fungi, ciliates, and green algae but not in animals or plants (Bochtler and Fernandes, 2021). Kong et al. (2022). developed a metagenomic method (a machine learning algorithm) and found that commensal or soil bacteria could explain the majority of 6 mA in insect and plant samples, and there is no evidence of the high 6 mA abundance in *Drosophila, Arabidopsis,* or humans. They also reported that even *E. coli plasmids* with Dam methyltransferase mutations were 6 mA-enriched, thus interfering with the evaluation of possible 6 mA methyltransferases and demethylases. Some researchers are optimistic about the existence of 6 mA. Using contamination-free UHPLC-MS/MS technology, they reported the presence of 6 mA in 3 cultured human cell lines (HEK293T, human mesenchymal stem cells, and human ESCs) without mycoplasma contamination (Liu et al., 2020).

### DNA Methylation and Demethylation of 6 mA

In eukaryotic DNA methylation and demethylation, “writers” (methyltransferase), “erasers” (demethylases), and “readers” (recognition protein) play central roles. An in-depth study of these three enzymes contributes to revealing the epigenetic mechanism of methylation modification. Here, we discuss 6 mA methyltransferases and demethylases and summarize the candidate proteins recognizing 6 mA that have been discovered thus far (Figure 2B).

### “Writers”-Methyltransferases of 6 mA

The methyl groups of 5 mC and 6 mA are catalyzed by methyltransferases *via* S-adenosylmethionine (SAM). 5 mC is formed by two kinds of methyltransferases to establish and maintain 5 mC formation together. For example, in mice and humans, Dnmt3 and Dnmt1 are responsible for the establishment and maintenance of 5 mC, respectively (Chen and Zhang, 2020). They add the methyl group on SAM to the fifth position of the cytosine ring, forming 5 mC methylation. Notably, in other organisms, their methyltransferases are mostly homologous with these two enzymes, such as MET1 and DRM2 in plants, and Dnmt5 and Dnmt1 in fungi (Schmitz et al., 2019).

For 6 mA, the methyl group on SAM was added to the sixth position of the adenine ring primarily with the help of the MT-70 methyltransferase family (Li et al., 2019; Joshi et al., 2021; Boulias and Greer, 2022). MT-A70 was considered to have evolved from M.MunI-like DNA 6 mA methyltransferases of bacteria (Wang et al., 2019). The 6 mA methyltransferases reported in eukaryotes are mainly members of the MT-A70 family, such as methyltransferase like 4 (METTL4) in most mammals, DAMT-1 in *C. elegans*, TAMT-1 and MTA1c in *Tetrahymena thermophila,* and DAMT in *Phytophthora* (a kind of fungi) (Figure 2B) (Greer et al., 2015; Luo et al., 2018b; Chen et al., 2018; Hao et al., 2020). Greer et al. indicated that DAMT-1 was a 6 mA methyltransferase in *C. elegans.* The evidence showed that 6 mA was significantly decreased after knocking down DAMT-1, which suppressed the transgenerational phenotypes, and the mutation of DPPW (the catalytic domain of DAMT-1) inhibited the increase in 6 mA (Greer et al., 2015). In *Thermophila ciliates,* MTA1c (a complex of MTA1, MTA9, p1, and p2) was reported as a 6 mA methyltransferase and had a special favor for the ApT context. In addition, Beh et al. (2019). found that MAT1 and MAT9 did not have the domain necessary for binding to DNA. Only in the presence of p1 and p2 can MTA1c catalyze 6 mA methylation. In *T. thermophila*, Wang et al. (2019). reported a methyltransferase named AMT1*,* which contained the catalytic motif DPPW. In rice, DDM1 played an important role in 6 mA methylation, and its mutations affected the development of rice by downregulating the expression levels of *GHD7*, *BRD1*, and *DWF7* (Zhang et al., 2018b). In *Phytophthora*, it was reported that the 6 mA level was significantly reduced and there was a greater loss in the second peak of the bimodal methylation pattern around the TSS in the DAMT mutant, which suggested that DAMT might contribute to 6 mA modification and prefer to the methylation gene bodies after the TSS (Chen et al., 2018). In almost all EDFs, including AMF, AMT1 was found to be a methyltransferase, and the ApT context was symmetrically methylated in the genome (Chaturvedi et al., 2021).

In mammals, the presence of 6 mA methyltransferase is controversial. In Mettl4 knockout (KO) mouse ESCs, the abundance of 6 mA dropped from an average of 8.6 p.p.m. in wild-type (WT) ESCs to an undetectable level (Kweon et al., 2019). The level of 6 mA in spleen genomic DNA also decreased with the inactivation of METTL4, which contains the catalytic motif DPPW. Furthermore, METTL4 was discovered to accumulate in mitochondria and suppress transcription at the mitochondrial promoter region by regulating 6 mA. In contrast, Chen et al. (2020). could not detect 6 mA in 293T cells, and alterations in METTL4 expression levels did not affect 6 mA detection. N-6 adenine-specific DNA methyltransferase 1 (N6AMT1) was reported as a methyltransferase in humans, containing a catalytic conserved motif NPPY (Xiao et al., 2018). The study indicated that silencing or overexpressing N6AMT1 could regulate the level of 6 mA in the human genome. However, another study found that N6AMT1 cannot function as a methyltransferase in glioblastoma (Xie et al., 2018). Structural analysis showed that N6AMT1 has the potential ability to catalyze adenine methylation in DNA. Nevertheless, the negative charges surrounding the active site make it difficult to bind to the negatively charged phosphate backbone of a DNA substrate (Li et al., 2019; Woodcock et al., 2019). One possible explanation is that N6AMT1 can bind to DNA in combination with some kind of partner proteins; however, such a hypothesis has not been confirmed in eukaryotes.

Remarkably, new evidence indicated that 6 mA modification in mammalian DNA is not methyltransferase-generated but DNA polymerase dependent. Musheev et al. (2020). showed that 6 mA was not dependent on methyltransferases but was incorporated by DNA polymerases, and one source of 6 mA may be m6A in RNA. Another study revealed that DNA polymerase lambda (Pol λ) contributed to 6 mA modification in DNA via nonhomologous end joining (NHEJ) repair (Liu et al., 2021a). The complex origins of 6 mA in mammals are not fully understood.

### “Erasers”-Demethylases of 6 mA

The removal of 5 mC is a classic demethylation process. The multistep erasure of 5 mC relies on the oxidation and removal of multiple methylation enzymes. A typical example is the demethylation of 5 mC mediated by TET in mammals (Fritz and Papavasiliou, 2010; Young et al., 2015; Wu and Zhang, 2017). Under the catalysis of TET, Fe^2+^, O_2_, and α-KG oxidized 5 mC to 5 hmC, 5fC, and 5caC successively (Tahiliani et al., 2009; He et al., 2011; Ito et al., 2011). In addition, SIDML2 in tomatoes, ROS1 in *Arabidopsis*, and T7H in fungi were all reported to be involved in 5 mC demethylation (Li et al., 2015; Lang et al., 2017; Zhang et al., 2018a).

Regarding 6 mA, studies on demethylases are more in-depth in mammals and less in other eukaryotic organisms, especially in plants and fungi (Chachar et al., 2021). Its removal is primarily dependent on the alpha-ketoglutarate-dependent dioxygenase (AlkB) family, which contains conserved Fe^2+^ and 2OG (2-oxoglutarate, α-KG)-dependent dioxygenase domains. It was reported that ALKBH1 could convert 6 mA to N6-hydroxymethyladenine (6 hmA), and 6 hmA could spontaneously degrade to adenine and generate formaldehyde without the catalysis of ALKBH1 (Figure 2B) (Xiao et al., 2018; Zhang et al., 2020). This is different from the methylation of 5 hmC, which does not produce formaldehyde due to nucleophilic attacks, such as exogenous thiols (Liutkeviciute et al., 2009; Schiesser et al., 2013). HALKBLH1 in the human ALKBH (hALKBH) family contains Flip0 at the N-terminus, a nucleotide recognition cap (NRL) containing Flip1 and Flip2, and a catalytic center. Key amino acid residues in NRL potentially determine the specific recognition and demethylation of hALKBH (Tian et al., 2020). In *C. elegans,* Greer et al. (2015). indicated that the mutant NMAD-1 could decrease demethylation ability, which suggested a new kind of demethylase. The methyltransferase of the 6 mA signature called DPPW in NMAD-1 was important for substrate recognition and catalytic activity. It was shown that in NMAD-1 mutant worms, their fertility was inhibited across generations. Xiao et al. (2018). reported that the ALKBH family might also be involved in the demethylation of 6 mA in *C. elegans*. In addition to the AlkB family, the TET family also plays an important role in the removal of 6 mA. In *Drosophila,* DMAD (*Drosophila* DNA 6 mA demethylase), a member of the TET family, is involved in the demethylation of 6 mA. It was demonstrated that DMAD had a core catalytic domain called DSBH (double-stranded β-helix) fold present in all AlkB family members and specifically inhibited modification of 6 mA, which played an important role in promoting GSC (germline stem cell) differentiation and resulted in the loss of germ cells (Zhang et al., 2015).

ALKBH1 is a demethylase in humans and mice. It was reported that ALKBH1 could precisely regulate the 6 mA level in mouse ESCs (Wu et al., 2016). The results of Li et al. (2020). also revealed the role of ALKBH1 as a DNA demethylase in mice. In humans, the expression of *ALKBH1* influenced the prevalence of 6 mA (Xiao et al., 2018). Furthermore, the role of ALKBH1 in human mitochondria was identified. The level of mitochondrial 6 mA in ALKBH1-KO cells was slightly higher than that in ALKBH1-WT cells (Koh et al., 2018). In addition, the demethylation effect of ALKBH1 was also reported in glioblastoma. The preference of ALKBH1 was demonstrated by a pull-down assay and ALKBH1 ChIP-seq (Xie et al., 2018). However, some reports indicated that ALKBH1 knockout had no impact on 6 mA levels in mouse ESCs and HEK293T cells, implicating the complexity of DNA demethylation (Liu et al., 2016a; Liu et al., 2020). ALKBH4 is orthologous to DMAD (6 mA demethylase in *Drosophila*) and NMAD-1 (6 mA demethylase in *C. elegans*). Its potential role in DNA demethylation in mice has been reported (Kweon et al., 2019). However, more evidence is still needed to confirm its role as a 6 mA demethylase.

### “Readers”-Proteins Recognizing 6 mA

Proteins that specifically recognize 5 mC-methylated DNA have been identified in the last century, such as MeCP2, a polypeptide containing both the methyl-CpG binding domain (MBD) and transcriptional inhibition domain (TRD) in mammals (Hendrich and Bird, 1998).

He et al. (2019) *.* found a kind of protein in the Fox family called Jumu, which could recognize and bind 6 mA-modified DNA in *Drosophila* (Figure 2B). Jumu can regulate early embryogenesis by inducing 6 mA-labeled genes called *Zelda.* Zelda was reported to positively regulate a group of miRNAs in *Drosophila* embryos by binding to *cis*-regulatory enhancers and affecting the expression of transcriptional regulators thereafter (Fu et al., 2014). After the Jumu-mutated oocytes combined with normal sperm, 72% of the embryos failed to develop into larvae. However, a zygote combined with mutant sperm and normal oocytes can develop normally, and most dead mutant embryos do not show a segmentation phenotype. Their study demonstrated the importance of the 6 mA-binding protein for the regulation of biological activity. Similarly, single-stranded DNA-binding protein 1 (SSBP1), containing HNRNP and YTH domains, was also considered another protein recognizing 6 mA in human mitochondria, which preferentially binds to ssDNA along the heavy chain, consistent with the position of 6 mA enrichment. The presence of 6 mA decreased the melting temperature of dsDNA, thus collecting SSBP1 into the heavy chain of mitochondria. (Koh et al., 2018; Shen et al., 2022). In addition, under hypoxic stress, the abundance of 6 mA in mitochondrial DNA was significantly increased, thus promoting the inhibition of mtDNA transcription by repressing the binding of mitochondrial transcription factor A (TFAM) (Hao et al., 2020). During the differentiation of trophoblast stem cells in mice, the expression of 6 mA in SIDD was significantly increased, which obstructed the binding of SATB1 to chromatin (Li et al., 2020). Some scholars questioned whether 6 mA could actively repel SATB1 binding because the dramatic bending of the DNA helix inhibited the binding of SATB (Li et al., 2021a; Boulias and Greer, 2022). Although these reports have shown the existence of proteins recognizing 6 mA, the downstream process after recognition is still not fully understood.

### Function of 6 mA

5 mC has attracted much attention, and multiple biological functions of 5 mC have been demonstrated, including transcription suppression, transposon suppression, genomic imprinting, X chromosome inactivation, and epigenetic memory (Bird, 2002; Chen et al., 2016; Wu and Zhang, 2017). However, research on 6 mA is still limited. In this section, we summarize several widely recognized functions of 6 mA in eukaryotes.

### 6 mA and Gene Expression

In different organisms, the prevalence of 6 mA showed different effects on gene expression. It has been confirmed that 6 mA promotes gene expression in *Oxytricha* (Beh et al., 2019)*,* rice (Zhang et al., 2018b), *Chlamydomonas* (Fu et al., 2015; Mondo et al., 2017), and fungi(Mondo et al., 2017) but not in mammals (Wu et al., 2016). The 6 mA level in *Oxytricha* was decreased by mutating the methyltransferase MTA1; however, only a few genes were significantly altered. The genes with a lower or greater loss of 6 mA markers near the TSS in mutants had little change in transcription, which meant drastic changes in the 6 mA level had a low effect on the level of overall transcription across the genome (Beh et al., 2019). This may be because the MTA1 mutant did not completely eliminate 6 mA or other DNA methylation modes in the genome can sufficiently buffer genes from changes in transcription. Similarly, in fungi, 6 mA might promote the likelihood of gene expression, and the level of actual gene expression may be regulated independently to maintain the stability of genome transcription (Mondo et al., 2017). The level of 6 mA-modified genes in wild-type rice was significantly higher than that in mutant rice (Zhang et al., 2018b). The R2R3-MYB protein in *Arabidopsis*, one of the largest transcription factors in the MYB family, has a significantly reduced affinity when binding to 6 mA-modified DNA compared to unmodified DNA (Wang et al., 2020). In *Chlamydomonas*, 6 mA near the TSS region marks active transcription genes(Fu et al., 2015). Sheng et al. also reported that the change in the 6 mA level in TSS was correlated with the expression of highly differentially expressed genes (DEGs) (Sheng et al., 2021). Although many reports have shown that 6 mA can promote gene expression in various eukaryotes, there is evidence supporting that 6 mA blocks the transcription of mammalian genes. For instance, the accumulation of 6 mA located on the X chromosome and Chr13 in mammals and the 6 mA density of young full-length line-1 transposons affected the inhibition of gene expression levels (Wu et al., 2016). In another study of 6 mA in mammalian mitochondria, the transcription of heavy and light chains with 6 mA modification at the promoter region was also inhibited *in vitro* (Hao et al., 2020). However, 6 mA was considered a marker of actively transcribed genes in human liver tissues (Cui et al., 2022). For the mechanism of 6 mA affecting gene transcription, one possible explanation is regulating the combination of genes and their transcription factors. It was reported that the decrease in 6 mA in the promoter of BMP2 could enhance the binding of October4 (octamer-binding transcription factor 4) and then activate BMP2 transcription (Ouyang et al., 2021). The detailed process and relevant molecules remain to be further studied.

### 6 mA and Nucleosome

Research has shown that 6 mA can assist in nucleosome localization. In *Chlamydia*, 6 mA near TSS sites presents periodic distribution and distributes between the small bodies that connect the nucleus, which may help nucleosome localization. If the distance between the two adjacent 6 mA sites is longer than the length of a nucleosome, the nucleosome is likely to be located between the two adjacent 6 mA sites (Fu et al., 2015). In ciliates, 6 mA is directly detrimental to nucleosome occupancy in local, quantitative, and intrinsic features *in vivo* (Luo. et al., 2018b; Beh et al., 2019). Similarly, Wang et al. reported that 6 mA and nucleosome distributions downstream of TSS had two damped oscillations with periods of ∼200 bp but opposite phases (Wang et al., 2017; Wang et al., 2019). However, 6 mA did not exhibit the ability to affect nucleosome occupancy *in vitro* due to endogenous chromatin assembly factors (such as trans-acting factors), DNA sequences, and chromatin remodeling complexes (Wang et al., 2017; Beh et al., 2019). Another reason is that 6 mA can change the curvature and stiffness of DNA, which is not conducive to the formation of small nucleosomes (Luo et al., 2018b). The relationship between 6 mA and nucleosomes in the eukaryotes mentioned above is similar to the function of 5 mC (Huff and Zilberman, 2014; Wang et al., 2017). The dense 5 mC on DNA could alter the major and minor grooves and not facilitate the curvature of DNA within nucleosomes, which would make the nucleosomes unstable (Pérez et al., 2012; Jimenez-Useche et al., 2013). The results suggest that different types of methylation modifications may affect nucleosome location. 6 mA modification and nucleosome localization may also regulate gene transcription and thus participate in a series of processes in eukaryotes. In starved *T. thermophila*, the amplitude (peak-to-trough distance) of nucleosome distribution was increased, whereas the amplitude of 6 mA distribution was decreased. This was probably because DNA replication and transcription perturbed nucleosomes. More highly methylated 6 mA sites were found in linker DNA, which could reinforce nucleosome stacking and stabilize it (Sheng et al., 2021). This suggests that the interaction of 6 mA and nucleosomes may play an important role in epigenetic processes. It was also reported that the decrease in 6 mA in the BMP2 promoter could promote the binding of October4 (octamer-binding transcription factor 4) to the BMP2 promoter and then increase BMP2 transcription (Ouyang et al., 2021). In another study, 6 mA was reported to promote heterochromatin formation in human glioblastoma *via* H3K9me3 histone modification (Xie et al., 2018). The effect of this relationship between 6 mA and nucleosome localization on gene transcription requires further in-deep research.

### 6 mA and Stress

Under the influence of 6 mA, eukaryotes have different tolerances to environmental stresses. In *Tetrahymena,* the global level of 6 mA was reduced, and the percentage of highly asymmetric 6 mA was increased under starvation (from 0.18% to 1.45% in vegetative cells and 0.12%–0.93% in starved cells). As mentioned above, upon starvation, the change in 6 mA located 1 kb downstream of TSS was correlated with the expression of DEGs (log2-fold change), and the nucleosome positioning degree was also increased in starved cells (Sheng et al., 2021). In rice, dysfunction of heat shock transcription factor A1 (HsfA1) and heat shock protein 70 (HSP 70) induced by 6 mA modifications decreases the sensitivity to heat stress. In addition, the increase in 6 mA density led to a decrease in cold resistance and increased salt and heat resistance (Zhang et al., 2018b). Under hypoxia, METTL4 was upregulated in mitochondria, leading to upregulation of 6 mA levels. This may be regulated by HIF1α and balance the increased ROS to adapt to hypoxia in mammals. (Hao et al., 2020). The hypoxic stress-induced HIF pathway may play an important role in human diseases (Jain et al., 2018; Guo et al., 2020; Liu et al., 2021b; Liu et al., 2022).

In addition, changes in mammalian environmental stress can cause changes in 6 mA, which means that neuronal activities may affect the prevalence and abundance of 6 mA. Evidence shows that 6 mA exists in the mammalian brain and increases upon stress, which is negatively correlated with LINE transposon expression. In the prefrontal cortex (PFC), 6 mA significantly accumulated and underwent dynamic changes upon chronic stress exposure. A negative correlation between 6 mA and the expression of some neuronal genes was also reported(Yao et al., 2017). Consistent with this, another group found that 6 mA was upregulated and negatively correlated with Hrt2a gene expression in the amygdala upon early life stress in rats (Kigar et al., 2017). Under hypoxic conditions, after ALKBH1 knockdown, genes in the hypoxia pathway were downregulated, and DNA damage and p53 pathway genes were upregulated in glioblastoma (Xie et al., 2018). However, which factors and pathways regulate gene expression changes under hypoxic conditions has not been discussed.

### 6 mA and Embryogenesis

6 mA may play an important role in embryonic development. The dynamic change was observed in the embryonic stage of *Drosophila*, and it may be regulated by DMAD, whose overexpression led to the loss of germ cells, including GSCs. This finding supported that DMAD may play a role in promoting GSC differentiation. (Zhang et al., 2015). Recently, evidence indicated that 6 mA was possibly related to mammalian embryogenesis. As mentioned earlier, the 6 mA density in pig embryos rose to ∼0.17% and then decreased to ∼0.05% during embryogenesis, suggesting the possible biological function of 6 mA (Liu et al., 2016b). In zebrafish embryos, the level of 6 mA increased to a maximum of ∼0.1% and then gradually decreased to approximately 0.006% (Liu et al., 2016b). However, another study showed that the level of 6 mA presented a linear increase in the embryonic states of mice and zebrafish (Fernandes et al., 2021). In mice, 6 mA is most abundant in the lungs, spleen, and brain, especially in the prefrontal cortex (PFC); therefore, it may play an important role in regulating the development of the nervous system and may be associated with certain neurological disorders (Fernandes et al., 2021). 6 mA was also detected in mouse ESCs. The authors found that 6 mA accumulated in the young long interspersed element 1 (LINE-1) and blocked transcription of their neighboring genes (Wu et al., 2016). Consistent with this conclusion, Li et al. (2020). also reported dynamic changes in 6 mA during early embryogenesis. The evidence showed that 6 mA mainly existed in intergenic regions, such as LINE-1s and modulated the ESC-to-TSC (trophoblast stem cell) transition by antagonizing SATB1 (a well-known SIDD regulating protein expressed in TSC).

### 6 mA and Human Disease

The extent of DNA methylation is related to the pathogenesis and progression of many diseases. 5 mC modification of DNA is closely related to hypertension (Han et al., 2016). Recently, the relationship between 6 mA and hypertension has also been revealed (Guo et al., 2020). In human and mouse hypertension models, leukocyte 6 mA DNA level was significantly decreased and returned to normal after successful treatment. The prevalence of 6 mA can regulate the expression of key genes and modify cell functions, which accelerates the pathological progress of human diseases. The investigators demonstrated the potential protective role of ALKBH1-mediated 6 mA level in Ang II-induced vascular remodeling. The silencing of ALKBH1 increased the prevalence of 6 mA in VSMCs and inhibited Ang II-induced phenotypic transformation, proliferation, and migration of VSMCs, mediated by the HIF1α-dependent pathway (Guo et al., 2020). In another study of patients with chronic kidney disease (CKD) in the clinical setting, Ouyang et al. (2021). found that the 6 mA level of leukocytes decreased significantly as the severity of vascular calcification (VC) increased. In addition, the mRNA expression of ALKBH1 was significantly upregulated in patients with CKD with VC, which could cause the change of 6 mA level in leukocytes(Chaudhary, 2022). The possible mechanism is that ALKBH1 reduces 6 mA density in the BMP2 promoter of VSMCs and thus promotes the binding of October4. BMP2 transcription is activated and induces an increase in RUNX2 expression thereafter, ultimately resulting in osteogenic reprogramming of VSMCs and VC progression. Using October4-knockout mice, they found that October4 could downregulate BMP2 expressions which could alleviate calcification effect of ALKBH1 overexpression (Rong et al., 2014; Ouyang et al., 2021). Another study reported that ALKBH1 promoted adipogenic differentiation and contributed to the accumulation of adipose tissue. The results showed that ALKBH1 decreased the 6 mA levels of HIF-1α and GYS1 and then activated the HIF-1 pathway (Liu et al., 2022).

Abnormal dynamic regulation of 6 mA has been reported in many cancers. 6 mA methyltransferases such as N6AMT1 have been shown to inhibit tumor progression (Xiao et al., 2018; Shen et al., 2022). A recent study showed that the density of 6 mA in highly expressed genes was significantly higher, and the 6 mA density was decreased in LINE and SINE gene repetition regions in HCC, which might lead to chromosome defects or rearrangements similar to 5 mC, thus promoting the development of cancer (Cui et al., 2022). However, how 6 mA affects subsequent biological processes has not been reported and is worth further investigation. Xiao et al. reported that 6 mA contents were decreased in primary gastric and liver cancers. Loss of 6 mA promoted tumorigenesis, which was related to the regulation of N6AMT1 and ALKBH1 (Xiao et al., 2018). Similarly, depletion of 6 mA led to the accumulation of sensor proteins such as ASXL1, which contributed to the onset and metastasis of aggressive tumors (Kweon et al., 2019). In glioblastoma, the dynamic regulation of 6 mA was related to tumor progression. The regulation of 6 mA methylation at specific sites by methyltransferases and demethylases has an impact on the proliferation, self-renewal, and formation capacity of tumors (Xie et al., 2018). In the occurrence of triple-negative breast cancer (TNBC), overexpression of *ALKBH1* or downregulation of *N6AMT1* can reduce the resistance of TNBC cells to olaparib (a PARP inhibitor targeting DNA repair). This may be due to the decreased level of 6 mA can reduce the expression of *LINP1*. Meanwhile, the overexpression of γ-H2AX (a marker of DNA damage) regulated by N6AMT1 in TNBC cells was significantly reduced, suggesting that 6 mA plays an important role in DNA damage repair (Sheng et al., 2020). Notably, intratumor bacteria were discovered in many human solid tumors (Nejman et al., 2020). Therefore, it is necessary to avoid possible bacterial contamination while detecting 6 mA in the tumor genome.

## Discussion

6 mA plays an important biological role in prokaryotes. Although many studies have indicated the presence of 6 mA in eukaryotes, bacterial contamination and other false positives of nonspecific methylation of DNA or RNA are still the primary factors affecting the prevalence and even the actual presence of 6 mA in eukaryotes. Therefore, it is necessary to use cross-validation of different detection methods to guarantee accuracy. Some methods to minimize the error were also proposed. To test bacterial contamination, amplification of prokaryotic 16 S rRNA genes by PCR using universal 16 S primers is recommended (Liang et al., 2020). Digesting RNA may also be taken into consideration to decrease interference. In the future, we hope more research will focus on developing a new generation of detection techniques that can exclude bacterial contamination and address false positives. In addition, existing publications need to be re-evaluated to determine 6 mA abundance and actual enzymes involved in 6 mA methylation, demethylation and cognition.

The incredibly low abundance of 6 mA reported in eukaryotes raises questions about its biological functions. The abundance of 6 mA presents dynamic changes during embryogenesis and under environmental stress. In addition, it varies among eukaryotes and even in different tissues and cells of the same organism. The large variation in 6 mA abundance among different reports is possibly due to bacterial contamination and different detection methods, or it may be related to the types, development stages, and nutritional status of cells. The level of 6 mA and the underlying factors that influence it need further confirmation. Importantly, researchers need to take action to prevent the results from interfering with bacterial contamination.

The enzymes of the MT-A70 family are common methyltransferases in eukaryotes. Recently, new evidence has shown that 6 mA is DNA polymerase-dependent (discussed earlier). Researchers have suggested that 6 mA plays a role in minimizing the incorporation of 8-oxo-2′-deoxyguanosine (8-oxoG) opposite to adenine by DNA polymerases and thus contributes to DNA damage repair based on the existing evidence. It is a noteworthy hypothesis, and we look forward to witnessing more promising discoveries in this direction. Demethylase has been studied extensively in metazoans but is relatively rare in plants and fungi. Under the catalysis of the demethylase ALKBH1, 6 mA is oxidized to 6 hmA, which can spontaneously degrade to adenine and generate formaldehyde without the catalysis of demethylase. However, the specific processes of other demethylases remain to be explored. In addition, there has been little focus on 6 mA binding proteins, and their potential function may be underestimated. Proteins that recognize 6 mA may assist methyltransferase and demethylase without domains that recognize 6 mA-modified DNA to regulate the expression of 6 mA and may have dramatic effects on various biological processes. In conclusion, the prevalence of 6 mA in eukaryotes is regulated by methyltransferases and demethylases; however, the existing studies on factors and pathways involving the process are limited. In addition, whether there are other enzymes that synergistically mediate the abundance of 6 mA in eukaryotes reminds to be explored. Recently, the relationship between 6 mA regulated by the methylase N6AMT1 and demethylase ALKBH1 and the occurrence of human diseases has been reported, which leads to a new research boom. We expect to see more breakthroughs in “writers,” “erasers,” and “readers” and shed light on the dynamics and roles of 6 mA in living organisms in the future.

Interestingly, dynamic changes in 6 mA abundance and specific enrichment of 6 mA suggest a link between 6 mA modification and specific biological processes, such as gene expression, nucleosome localization, stress, development of embryogenesis, and human diseases. 6 mA may promote the expression of modified genes; however, in some eukaryotes, the overall transcription level may remain stable due to an independent regulatory mechanism. The same period and opposite phases between 6 mA and nucleosome suggest that the interaction of 6 mA and nucleosome may play an important role in epigenetic processes. In starved *T. thermophila*, the amplitude (peak-to-trough distance) of nucleosome distribution was increased, whereas the amplitude of 6 mA distribution was decreased. This was probably because DNA replication and transcription perturbed nucleosomes, which demonstrated that 6 mA played an important role in eukaryotic metabolic processes and cellular pathways. However, because of technical limitations and possible bacterial contamination, these results need to be treated with caution. In addition, in response to stress and pathological factors resulting in human diseases, the factors regulating 6 mA level and related pathways should be the focus of future research. For humans, we can explore more potential roles of 6 mA, such as being a marker for the development of certain diseases, a target for certain tumors, or a prognosis for certain diseases.

The path of science is fraught with controversy. Owing to the low density and bacterial contamination of 6 mA in eukaryotes, the function of 6 mA eukaryotes has not been accepted until recently. Debates are continuing regarding the presence of 6 mA modification of DNA in eukaryotes. In eukaryotes, the study of 6 mA has just entered the initial stage. As further experimentation and profound discussion are being conducted in this emerging field, the full picture of 6 mA in mammals will be uncovered.

## References

[B1] Beh L. Y. Debelouchina G. T. Clay D. M. Thompson R. E. Lindblad K. A. Hutton E. R. (2019). Identification of a DNA N6-Adenine Methyltransferase Complex and its Impact on Chromatin Organization. Cell. 177 (7), 1781–1796. 10.1016/j.cell.2019.04.028 31104845 PMC6570567

[B2] Bewick A. J. Hofmeister B. T. Powers R. A. Mondo S. J. Grigoriev I. V. James T. Y. (2019). Diversity of Cytosine Methylation across the Fungal Tree of Life. Nat. Ecol. Evol. 3 (3), 479–490. 10.1038/s41559-019-0810-9 30778188 PMC6533610

[B3] Bird A. (2002). DNA Methylation Patterns and Epigenetic Memory. Genes. Dev. 16 (1), 6–21. 10.1101/gad.947102 11782440

[B4] Bochtler M. Fernandes H. (2021). DNA Adenine Methylation in Eukaryotes: Enzymatic Mark or a Form of DNA Damage? BioEssays 43 (3), 2000243. 10.1002/bies.202000243 33244833

[B5] Boulias K. Greer E. L. (2022). Means, Mechanisms and Consequences of Adenine Methylation in DNA. Nat. Rev. Genet. 10.1038/s41576-022-00456-x PMC9354840 35256817

[B6] Cai J. Xiao G. Su R. (2022). GC6mA-Pred: A Deep Learning Approach to Identify DNA N6-Methyladenine Sites in the Rice Genome. Methods 204, 14–21. 10.1016/j.ymeth.2022.02.001 35149214

[B7] Chachar S. Liu J. Zhang P. Riaz A. Guan C. Liu S. (2021). Harnessing Current Knowledge of DNA N6-Methyladenosine from Model Plants for Non-model Crops. Front. Genet. 12, 668317. 10.3389/fgene.2021.668317 33995495 PMC8118384

[B8] Chaturvedi A. Cruz Corella J. Robbins C. Loha A. Menin L. Gasilova N. (2021). The Methylome of the Model Arbuscular Mycorrhizal Fungus, Rhizophagus Irregularis, Shares Characteristics with Early Diverging Fungi and Dikarya. Commun. Biol. 4 (1), 901. 10.1038/s42003-021-02414-5 34294866 PMC8298701

[B9] Chaudhary M. (2022). Novel Methylation Mark and Essential Hypertension. J. Genet. Eng. Biotechnol. 20 (1), 11. 10.1186/s43141-022-00301-y 35061109 PMC8777530

[B10] Chen H. Gu L. Orellana E. A. Wang Y. Guo J. Liu Q. (2020). METTL4 Is an snRNA m6Am Methyltransferase that Regulates RNA Splicing. Cell. Res. 30 (6), 544–547. 10.1038/s41422-019-0270-4 31913360 PMC7264358

[B11] Chen H. Shu H. Wang L. Zhang F. Li X. Ochola S. O. (2018). Phytophthora Methylomes Are Modulated by 6mA Methyltransferases and Associated with Adaptive Genome Regions. Genome Biol. 19 (1), 181. 10.1186/s13059-018-1564-4 30382931 PMC6211444

[B12] Chen K. Zhao B. S. He C. (2016). Nucleic Acid Modifications in Regulation of Gene Expression. Cell. Chem. Biol. 23 (1), 74–85. 10.1016/j.chembiol.2015.11.007 26933737 PMC4779186

[B13] Chen Z. Zhang Y. (2020). Role of Mammalian DNA Methyltransferases in Development. Annu. Rev. Biochem. 89, 135–158. 10.1146/annurev-biochem-103019-102815 31815535

[B14] Cui H. Rong W. Ma J. Zhu Q. Jiang B. Zhang L. (2022). DNA N6-Adenine Methylation in HBV-Related Hepatocellular Carcinoma. Gene 822, 146353. 10.1016/j.gene.2022.146353 35189250

[B15] Douvlataniotis K. Bensberg M. Lentini A. Gylemo B. Nestor C. E. (2020). No Evidence for DNA N 6 -methyladenine in Mammals. Sci. Adv. 6 (12), eaay3335. 10.1126/sciadv.aay3335 32206710 PMC7080441

[B16] Feng S. Cokus S. J. Zhang X. Chen P.-Y. Bostick M. Goll M. G. (2010). Conservation and Divergence of Methylation Patterning in Plants and Animals. Proc. Natl. Acad. Sci. U.S.A. 107 (19), 8689–8694. 10.1073/pnas.1002720107 20395551 PMC2889301

[B17] Fernandes S. B. Grova N. Roth S. Duca R. C. Godderis L. Guebels P. (2021). N6-Methyladenine in Eukaryotic DNA: Tissue Distribution, Early Embryo Development, and Neuronal Toxicity. Front. Genet. 12, 657171. 10.3389/fgene.2021.657171 34108991 PMC8181416

[B18] Flusberg B. A. Webster D. R. Lee J. H. Travers K. J. Olivares E. C. Clark T. A. (2010). Direct Detection of DNA Methylation during Single-Molecule, Real-Time Sequencing. Nat. Methods 7 (6), 461–465. 10.1038/nmeth.1459 20453866 PMC2879396

[B19] Fritz E. L. Papavasiliou F. N. (2010). Cytidine Deaminases: AIDing DNA Demethylation? Genes. Dev. 24 (19), 2107–2114. 10.1101/gad.1963010 20889711 PMC2947763

[B20] Fu S. Nien C.-Y. Liang H.-L. Rushlow C. (2014). Co-activation of microRNAs by Zelda Is Essential for Early Drosophila Development. Dev. Camb. Engl. 141 (10), 2108–2118. 10.1242/dev.108118 PMC4011091 24764079

[B21] Fu Y. Luo G.-Z. Chen K. Deng X. Yu M. Han D. (2015). N6-methyldeoxyadenosine Marks Active Transcription Start Sites in Chlamydomonas. Cell. 161 (4), 879–892. 10.1016/j.cell.2015.04.010 25936837 PMC4427561

[B22] Greer E. L. Blanco M. A. Gu L. Sendinc E. Liu J. Aristizábal-Corrales D. (2015). DNA Methylation on N6-Adenine in *C. elegans* . Cell. 161 (4), 868–878. 10.1016/j.cell.2015.04.005 25936839 PMC4427530

[B23] Guo Y. Pei Y. Li K. Cui W. Zhang D. (2020). DNA N6-Methyladenine Modification in Hypertension. Aging 12 (7), 6276–6291. 10.18632/aging.103023 32283543 PMC7185115

[B24] Han L. Liu Y. Duan S. Perry B. Li W. He Y. (2016). DNA Methylation and Hypertension: Emerging Evidence and Challenges. Brief. Funct. Genomics 15 (6), 460–469. 10.1093/bfgp/elw014 27142121

[B25] Hao Z. Wu T. Cui X. Zhu P. Tan C. Dou X. (2020). N6-Deoxyadenosine Methylation in Mammalian Mitochondrial DNA. Mol. Cell 78 (3), 382–395. 10.1016/j.molcel.2020.02.018 32183942 PMC7214128

[B26] Hasan M. M. Basith S. Khatun M. S. Lee G. Manavalan B. Kurata H. (2021). Meta-i6mA: an Interspecies Predictor for Identifying DNA N6-Methyladenine Sites of Plant Genomes by Exploiting Informative Features in an Integrative Machine-Learning Framework. Briefings Bioinforma. 22 (3), bbaa202. 10.1093/bib/bbaa202 32910169

[B27] He S. Zhang G. Wang J. Gao Y. Sun R. Cao Z. (2019). 6mA-DNA-binding Factor Jumu Controls Maternal-To-Zygotic Transition Upstream of Zelda. Nat. Commun. 10 (1), 2219. 10.1038/s41467-019-10202-3 31101825 PMC6525185

[B28] He Y.-F. Li B.-Z. Li Z. Liu P. Wang Y. Tang Q. (2011). Tet-mediated Formation of 5-carboxylcytosine and its Excision by TDG in Mammalian DNA. Science 333 (6047), 1303–1307. 10.1126/science.1210944 21817016 PMC3462231

[B29] Hendrich B. Bird A. (1998). Identification and Characterization of a Family of Mammalian Methyl-CpG Binding Proteins. Mol. Cell. Biol. 18 (11), 6538–6547. 10.1128/mcb.18.11.6538 9774669 PMC109239

[B30] Huff J. T. Zilberman D. (2014). Dnmt1-independent CG Methylation Contributes to Nucleosome Positioning in Diverse Eukaryotes. Cell. 156 (6), 1286–1297. 10.1016/j.cell.2014.01.029 24630728 PMC3969382

[B31] Ito S. Shen L. Dai Q. Wu S. C. Collins L. B. Swenberg J. A. (2011). Tet Proteins Can Convert 5-methylcytosine to 5-formylcytosine and 5-carboxylcytosine. Science 333 (6047), 1300–1303. 10.1126/science.1210597 21778364 PMC3495246

[B32] Jain T. Nikolopoulou E. A. Xu Q. Qu A. (2018). Hypoxia Inducible Factor as a Therapeutic Target for Atherosclerosis. Pharmacol. Ther. 183, 22–33. 10.1016/j.pharmthera.2017.09.003 28942242

[B33] Jeong H. M. Lee S. Chae H. Kim R. Kwon M. J. Oh E. (2016). Efficiency of Methylated DNA Immunoprecipitation Bisulphite Sequencing for Whole-Genome DNA Methylation Analysis. Epigenomics 8 (8), 1061–1077. 10.2217/epi-2016-0038 27266718

[B34] Jimenez-Useche I. Ke J. Tian Y. Shim D. Howell S. C. Qiu X. (2013). DNA Methylation Regulated Nucleosome Dynamics. Sci. Rep. 3, 2121. 10.1038/srep02121 23817195 PMC3698496

[B35] Joshi S. Ujaoney A. K. Ghosh P. Deobagkar D. D. Basu B. (2021). N6-methyladenine and Epigenetic Immunity of Deinococcus Radiodurans. Res. Microbiol. 172 (1), 103789. 10.1016/j.resmic.2020.10.004 33188877

[B36] Khanal J. Lim D. Y. Tayara H. Chong K. T. (2021). i6mA-stack: A Stacking Ensemble-Based Computational Prediction of DNA N6-Methyladenine (6mA) Sites in the Rosaceae Genome. Genomics 113 (1 Pt 2), 582–592. 10.1016/j.ygeno.2020.09.054 33010390

[B37] Kigar S. L. Chang L. Guerrero C. R. Sehring J. R. Cuarenta A. Parker L. L. (2017). N6-methyladenine Is an Epigenetic Marker of Mammalian Early Life Stress. Sci. Rep. 7 (1), 18078. 10.1038/s41598-017-18414-7 29273787 PMC5741724

[B38] Koh C. W. Q. Goh Y. T. Toh J. D. W. Neo S. P. Ng S. B. Gunaratne J. (2018). Single-nucleotide-resolution Sequencing of humanN6-Methyldeoxyadenosine Reveals Strand-Asymmetric Clusters Associated with SSBP1 on the Mitochondrial Genome. Nucleic acids Res. 46 (22), 11659–11670. 10.1093/nar/gky1104 30412255 PMC6294517

[B39] Kong Y. Cao L. Deikus G. Fan Y. Mead E. A. Lai W. (2022). Critical Assessment of DNA Adenine Methylation in Eukaryotes Using Quantitative Deconvolution. Science 375 (6580), 515–522. 10.1126/science.abe7489 35113693 PMC9382770

[B40] Kweon S.-M. Chen Y. Moon E. Kvederaviciutė K. Klimasauskas S. Feldman D. E. (2019). An Adversarial DNA N6-Methyladenine-Sensor Network Preserves Polycomb Silencing. Mol. Cell 74 (6), 1138–1147. 10.1016/j.molcel.2019.03.018 30982744 PMC6591016

[B41] Laird P. W. (2010). Principles and Challenges of Genome-wide DNA Methylation Analysis. Nat. Rev. Genet. 11 (3), 191–203. 10.1038/nrg2732 20125086

[B42] Lang Z. Wang Y. Tang K. Tang D. Datsenka T. Cheng J. (2017). Critical Roles of DNA Demethylation in the Activation of Ripening-Induced Genes and Inhibition of Ripening-Repressed Genes in Tomato Fruit. Proc. Natl. Acad. Sci. U.S.A. 114 (22), E4511–E4519. 10.1073/pnas.1705233114 28507144 PMC5465898

[B43] Li W. Shi Y. Zhang T. Ye J. Ding J. (2019). Structural Insight into Human N6amt1-Trm112 Complex Functioning as a Protein Methyltransferase. Cell. Discov. 5, 51. 10.1038/s41421-019-0121-y 31636962 PMC6796863

[B44] Li W. Zhang T. Ding J. (2015). Molecular Basis for the Substrate Specificity and Catalytic Mechanism of Thymine-7-Hydroxylase in Fungi. Nucleic Acids Res. 43 (20), gkv979–10038. 10.1093/nar/gkv979 PMC4787775 26429971

[B45] Li X. Zhang Z. Luo X. Schrier J. Yang A. D. Wu T. P. (2021a). The Exploration of N6-Deoxyadenosine Methylation in Mammalian Genomes. Protein Cell. 12 (10), 756–768. 10.1007/s13238-021-00866-3 34405377 PMC8464638

[B46] Li Z. Jiang H. Kong L. Chen Y. Lang K. Fan X. (2021b). Deep6mA: A Deep Learning Framework for Exploring Similar Patterns in DNA N6-Methyladenine Sites across Different Species. PLoS Comput. Biol. 17 (2), e1008767. 10.1371/journal.pcbi.1008767 33600435 PMC7924747

[B47] Li Z. Zhao S. Nelakanti R. V. Lin K. Wu T. P. Alderman M. H. (2020). N6-methyladenine in DNA Antagonizes SATB1 in Early Development. Nature 583 (7817), 625–630. 10.1038/s41586-020-2500-9 32669713 PMC8596487

[B48] Liang Z. Riaz A. Chachar S. Ding Y. Du H. Gu X. (2020). Epigenetic Modifications of mRNA and DNA in Plants. Mol. plant 13 (1), 14–30. 10.1016/j.molp.2019.12.007 31863849

[B49] Liang Z. Shen L. Cui X. Bao S. Geng Y. Yu G. (2018). DNA N-Adenine Methylation in *Arabidopsis thaliana* . Dev. Cell 45 (3), 406–416. 10.1016/j.devcel.2018.03.012 29656930

[B50] Liu B. Liu X. Lai W. Wang H. (2017). Metabolically Generated Stable Isotope-Labeled Deoxynucleoside Code for Tracing DNA N6-Methyladenine in Human Cells. Anal. Chem. 89 (11), 6202–6209. 10.1021/acs.analchem.7b01152 28471639

[B51] Liu B. Wang H. (2021). Detection of N6-Methyladenine in Eukaryotes. Adv. Exp. Med. Biol. 1280, 83–95. 10.1007/978-3-030-51652-9_6 33791976

[B52] Liu F. Clark W. Luo G. Wang X. Fu Y. Wei J. (2016a). ALKBH1-Mediated tRNA Demethylation Regulates Translation. Cell. 167 (3), 816–828. 10.1016/j.cell.2016.09.038 27745969 PMC5119773

[B53] Liu J. Zhu Y. Luo G.-Z. Wang X. Yue Y. Wang X. (2016b). Abundant DNA 6mA Methylation during Early Embryogenesis of Zebrafish and Pig. Nat. Commun. 7, 13052. 10.1038/ncomms13052 27713410 PMC5059759

[B54] Liu X. Lai W. Li Y. Chen S. Liu B. Zhang N. (2020). N6-methyladenine Is Incorporated into Mammalian Genome by DNA Polymerase. Cell. Res. 31, 94–97. 10.1038/s41422-020-0317-6 32355286 PMC7853133

[B55] Liu X. Lai W. Li Y. Chen S. Liu B. Zhang N. (2021a). N6-methyladenine Is Incorporated into Mammalian Genome by DNA Polymerase. Cell. Res. 31 (1), 94–97. 10.1038/s41422-020-0317-6 32355286 PMC7853133

[B56] Liu X. Xie P. Hao N. Zhang M. Liu Y. Liu P. (2021). HIF-1-regulated Expression of Calreticulin Promotes Breast Tumorigenesis and Progression through Wnt/β-Catenin Pathway Activation. Proc. Natl. Acad. Sci. U.S.A. 118 (44), 44118. 10.1073/pnas.2109144118 PMC8612225 34706936

[B57] Liu Y. Chen Y. Wang Y. Jiang S. Lin W. Wu Y. (2022). DNA Demethylase ALKBH1 Promotes Adipogenic Differentiation via Regulation of HIF-1 Signaling. J. Biol. Chem. 298 (1), 101499. 10.1016/j.jbc.2021.101499 34922943 PMC8760519

[B58] Liutkevičiūtė Z. Lukinavičius G. Masevičius V. Daujotytė D. Klimašauskas S. (2009). Cytosine-5-methyltransferases Add Aldehydes to DNA. Nat. Chem. Biol. 5 (6), 400–402. 10.1038/nchembio.172 19430486

[B59] Lizarraga A. O’Brown Z. K. Boulias K. Roach L. Greer E. L. Johnson P. J. (2020). Adenine DNA Methylation, 3D Genome Organization, and Gene Expression in the Parasite Trichomonas Vaginalis. Proc. Natl. Acad. Sci. U.S.A. 117 (23), 13033–13043. 10.1073/pnas.1917286117 32461362 PMC7293704

[B60] Luo C. Hajkova P. Ecker J. R. (2018a). Dynamic DNA Methylation: In the Right Place at the Right Time. Science 361 (6409), 1336–1340. 10.1126/science.aat6806 30262495 PMC6197482

[B61] Luo G.-Z. Hao Z. Luo L. Shen M. Sparvoli D. Zheng Y. (2018b). N6-methyldeoxyadenosine Directs Nucleosome Positioning in Tetrahymena DNA. Genome Biol. 19 (1), 200. 10.1186/s13059-018-1573-3 30454035 PMC6245762

[B62] Lv H. Dao F. Y. Zhang D. Yang H. Lin H. (2021). Advances in Mapping the Epigenetic Modifications of 5‐methylcytosine (5mC), N6‐methyladenine (6mA), and N4‐methylcytosine (4mC). Biotech Bioeng. 118 (11), 4204–4216. 10.1002/bit.27911 34370308

[B63] Ma C. Niu R. Huang T. Shao L.-W. Peng Y. Ding W. (2019). N6-methyldeoxyadenine Is a Transgenerational Epigenetic Signal for Mitochondrial Stress Adaptation. Nat. Cell. Biol. 21 (3), 319–327. 10.1038/s41556-018-0238-5 30510156

[B64] Martisova A. Holcakova J. Izadi N. Sebuyoya R. Hrstka R. Bartosik M. (2021). DNA Methylation in Solid Tumors: Functions and Methods of Detection. Ijms 22 (8), 4247. 10.3390/ijms22084247 33921911 PMC8073724

[B65] Mondo S. J. Dannebaum R. O. Kuo R. C. Louie K. B. Bewick A. J. LaButti K. (2017). Widespread Adenine N6-Methylation of Active Genes in Fungi. Nat. Genet. 49 (6), 964–968. 10.1038/ng.3859 28481340

[B66] Morgan R. D. Dwinell E. A. Bhatia T. K. Lang E. M. Luyten Y. A. (2009). The MmeI Family: Type II Restriction-Modification Enzymes that Employ Single-Strand Modification for Host Protection. Nucleic acids Res. 37 (15), 5208–5221. 10.1093/nar/gkp534 19578066 PMC2731913

[B67] Musheev M. U. Baumgärtner A. Krebs L. Niehrs C. (2020). The Origin of Genomic N6-Methyl-Deoxyadenosine in Mammalian Cells. Nat. Chem. Biol. 16 (6), 630–634. 10.1038/s41589-020-0504-2 32203414

[B68] Nejman D. Livyatan I. Fuks G. Gavert N. Zwang Y. Geller L. T. (2020). The Human Tumor Microbiome Is Composed of Tumor Type-specific Intracellular Bacteria. Science 368 (6494), 973–980. 10.1126/science.aay9189 32467386 PMC7757858

[B69] O’Brown Z. K. Boulias K. Wang J. Wang S. Y. O’Brown N. M. Hao Z. (2019). Sources of Artifact in Measurements of 6mA and 4mC Abundance in Eukaryotic Genomic DNA. BMC genomics 20 (1), 445. 10.1186/s12864-019-5754-6 31159718 PMC6547475

[B70] Ouyang L. Su X. Li W. Tang L. Zhang M. Zhu Y. (2021). ALKBH1-demethylated DNA N6-Methyladenine Modification Triggers Vascular Calcification via Osteogenic Reprogramming in Chronic Kidney Disease. J. Clin. investigation 131 (14), 46985. 10.1172/JCI146985 PMC8279589 34003800

[B71] Pérez A. Castellazzi C. L. Battistini F. Collinet K. Flores O. Deniz O. (2012). Impact of Methylation on the Physical Properties of DNA. Biophysical J. 102 (9), 2140–2148. 10.1016/j.bpj.2012.03.056 PMC3341543 22824278

[B72] Razin A. (1984). “DNA Methylation Patterns: Formation and Biological Functions,” in DNA Methylation: Biochemistry and Biological Significance. Editors Razin A. Cedar H. Riggs A. D. (New York, NY: Springer New York), 127–146. 10.1007/978-1-4613-8519-6_7

[B73] Rehman M. U. Chong K. T. (2020). DNA6mA-MINT: DNA-6mA Modification Identification Neural Tool. Genes. 11 (8), 898. 10.3390/genes11080898 PMC7463462 32764497

[B74] Reisenauer A. Kahng L. S. McCollum S. Shapiro L. (1999). Bacterial DNA Methylation: a Cell Cycle Regulator? J. Bacteriol. 181 (17), 5135–5139. 10.1128/jb.181.17.5135-5139.1999 10464180 PMC94015

[B75] Rodriguez F. Yushenova I. A. DiCorpo D. Arkhipova I. R. (2022). Bacterial N4-Methylcytosine as an Epigenetic Mark in Eukaryotic DNA. Nat. Commun. 13 (1), 1072. 10.1038/s41467-022-28471-w 35228526 PMC8885841

[B76] Rong S. Zhao X. Jin X. Zhang Z. Chen L. Zhu Y. (2014). Vascular Calcification in Chronic Kidney Disease Is Induced by Bone Morphogenetic Protein-2 via a Mechanism Involving the Wnt/-Catenin Pathway. Cell. Physiol. Biochem. 34 (6), 2049–2060. 10.1159/000366400 25562153

[B77] Schadt E. E. Banerjee O. Fang G. Feng Z. Wong W. H. Zhang X. (2013). Modeling Kinetic Rate Variation in Third Generation DNA Sequencing Data to Detect Putative Modifications to DNA Bases. Genome Res. 23 (1), 129–141. 10.1101/gr.136739.111 23093720 PMC3530673

[B78] Schiesser S. Pfaffeneder T. Sadeghian K. Hackner B. Steigenberger B. Schröder A. S. (2013). Deamination, Oxidation, and C-C Bond Cleavage Reactivity of 5-hydroxymethylcytosine, 5-formylcytosine, and 5-carboxycytosine. J. Am. Chem. Soc. 135 (39), 14593–14599. 10.1021/ja403229y 23980549

[B79] Schiffers S. Ebert C. Rahimoff R. Kosmatchev O. Steinbacher J. Bohne A.-V. (2017). Quantitative LC-MS Provides No Evidence for m6dA or m4dC in the Genome of Mouse Embryonic Stem Cells and Tissues. Angew. Chem. Int. Ed. 56 (37), 11268–11271. 10.1002/anie.201700424 28371147

[B80] Schmitz R. J. Lewis Z. A. Goll M. G. (2019). DNA Methylation: Shared and Divergent Features across Eukaryotes. Trends Genet. 35 (11), 818–827. 10.1016/j.tig.2019.07.007 31399242 PMC6825889

[B81] Shah K. Cao W. Ellison C. E. (2019). Adenine Methylation in Drosophila Is Associated with the Tissue-specific Expression of Developmental and Regulatory Genes. G3 (Bethesda, Md 9 (6), 1893–1900. 10.1534/g3.119.400023 PMC6553526 30988038

[B82] Shanmuganathan R. Basheer N. B. Amirthalingam L. Muthukumar H. Kaliaperumal R. Shanmugam K. (2013). Conventional and Nanotechniques for DNA Methylation Profiling. J. Mol. Diagnostics 15 (1), 17–26. 10.1016/j.jmoldx.2012.06.007 23127612

[B83] Shen C. Wang K. Deng X. Chen J. (2022). DNA N6-Methyldeoxyadenosine in Mammals and Human Disease. Trends Genet. 38, 454–467. 10.1016/j.tig.2021.12.003 34991904 PMC9007851

[B84] Sheng X. Wang J. Guo Y. Zhang J. Luo J. (2020). DNA N6-Methyladenine (6mA) Modification Regulates Drug Resistance in Triple Negative Breast Cancer. Front. Oncol. 10, 616098. 10.3389/fonc.2020.616098 33614498 PMC7887291

[B85] Sheng Y. Pan B. Wei F. Wang Y. Gao S. (2021). Case Study of the Response of N 6 -Methyladenine DNA Modification to Environmental Stressors in the Unicellular Eukaryote Tetrahymena Thermophila. mSphere 6 (3), e0120820. 10.1128/mSphere.01208-20 34047647 PMC8265677

[B86] Smith Z. D. Meissner A. (2013). DNA Methylation: Roles in Mammalian Development. Nat. Rev. Genet. 14 (3), 204–220. 10.1038/nrg3354 23400093

[B87] Song L. James S. R. Kazim L. Karpf A. R. (2005). Specific Method for the Determination of Genomic DNA Methylation by Liquid Chromatography-Electrospray Ionization Tandem Mass Spectrometry. Anal. Chem. 77 (2), 504–510. 10.1021/ac0489420 15649046

[B88] Tahiliani M. Koh K. P. Shen Y. Pastor W. A. Bandukwala H. Brudno Y. (2009). Conversion of 5-methylcytosine to 5-hydroxymethylcytosine in Mammalian DNA by MLL Partner TET1. Science 324 (5929), 930–935. 10.1126/science.1170116 19372391 PMC2715015

[B89] Tian L.-F. Liu Y.-P. Chen L. Tang Q. Wu W. Sun W. (2020). Structural Basis of Nucleic Acid Recognition and 6mA Demethylation by Human ALKBH1. Cell. Res. 30 (3), 272–275. 10.1038/s41422-019-0233-9 32051559 PMC7054395

[B90] Tsukiyama S. Hasan M. M. Deng H.-W. Kurata H. (2022). BERT6mA: Prediction of DNA N6-Methyladenine Site Using Deep Learning-Based Approaches. Briefings Bioinforma. 23, bbac053. 10.1093/bib/bbac053 PMC8921755 35225328

[B91] Usai G. Vangelisti A. Simoni S. Giordani T. Natali L. Cavallini A. (2021). DNA Modification Patterns within the Transposable Elements of the Fig (Ficus Carica L.) Genome. Plants 10 (3), 451. 10.3390/plants10030451 33673593 PMC7997441

[B92] Vasu K. Nagaraja V. (2013). Diverse Functions of Restriction-Modification Systems in Addition to Cellular Defense. Microbiol. Mol. Biol. Rev. 77 (1), 53–72. 10.1128/MMBR.00044-12 23471617 PMC3591985

[B93] Vogeser M. Seger C. (2008). A Decade of HPLC-MS/MS in the Routine Clinical Laboratory - Goals for Further Developments. Clin. Biochem. 41 (9), 649–662. 10.1016/j.clinbiochem.2008.02.017 18374660

[B94] Wang B. Luo Q. Li Y. Yin L. Zhou N. Li X. (2020). Structural Insights into Target DNA Recognition by R2R3-MYB Transcription Factors. Nucleic acids Res. 48 (1), 460–471. 10.1093/nar/gkz1081 31733060 PMC7145699

[B95] Wang Y. Chen X. Sheng Y. Liu Y. Gao S. (2017). N6-adenine DNA Methylation Is Associated with the Linker DNA of H2A.Z-Containing Well-Positioned Nucleosomes in Pol II-Transcribed Genes in Tetrahymena. Nucleic acids Res. 45 (20), 11594–11606. 10.1093/nar/gkx883 29036602 PMC5714169

[B96] Wang Y. Sheng Y. Liu Y. Zhang W. Cheng T. Duan L. (2019). A Distinct Class of Eukaryotic MT-A70 Methyltransferases Maintain Symmetric DNA N6-Adenine Methylation at the ApT Dinucleotides as an Epigenetic Mark Associated with Transcription. Nucleic acids Res. 47 (22), 11771–11789. 10.1093/nar/gkz1053 31722409 PMC7145601

[B97] Wang Y. Zhang P. Guo W. Liu H. Li X. Zhang Q. (2021). A Deep Learning Approach to Automate Whole‐genome Prediction of Diverse Epigenomic Modifications in Plants. New Phytol. 232 (2), 880–897. 10.1111/nph.17630 34287908

[B98] Wion D. Casadesús J. (2006). N6-methyl-adenine: an Epigenetic Signal for DNA-Protein Interactions. Nat. Rev. Microbiol. 4 (3), 183–192. 10.1038/nrmicro1350 16489347 PMC2755769

[B99] Woodcock C. B. Yu D. Zhang X. Cheng X. (2019). Human HemK2/KMT9/N6AMT1 Is an Active Protein Methyltransferase, but Does Not Act on DNA *In Vitro*, in the Presence of Trm112. Cell. Discov. 5, 50. 10.1038/s41421-019-0119-5 31632689 PMC6796829

[B100] Wu T. P. Wang T. Seetin M. G. Lai Y. Zhu S. Lin K. (2016). DNA Methylation on N6-Adenine in Mammalian Embryonic Stem Cells. Nature 532 (7599), 329–333. 10.1038/nature17640 27027282 PMC4977844

[B101] Wu X. Zhang Y. (2017). TET-mediated Active DNA Demethylation: Mechanism, Function and beyond. Nat. Rev. Genet. 18 (9), 517–534. 10.1038/nrg.2017.33 28555658

[B102] Xiao C.-L. Zhu S. He M. Chen D. Zhang Q. Chen Y. (2018). N6-Methyladenine DNA Modification in the Human Genome. Mol. Cell 71 (2), 306–318. 10.1016/j.molcel.2018.06.015 30017583

[B103] Xie Q. Wu T. P. Gimple R. C. Li Z. Prager B. C. Wu Q. (2018). N-methyladenine DNA Modification in Glioblastoma. Cell. 175 (5), 1228–1243. 10.1016/j.cell.2018.10.006 30392959 PMC6433469

[B104] Yang S. Wang Y. Chen Y. Dai Q. (2020). MASQC: Next Generation Sequencing Assists Third Generation Sequencing for Quality Control in N6-Methyladenine DNA Identification. Front. Genet. 11, 269. 10.3389/fgene.2020.00269 32269589 PMC7109398

[B105] Yao B. Cheng Y. Wang Z. Li Y. Chen L. Huang L. (2017). DNA N6-Methyladenine Is Dynamically Regulated in the Mouse Brain Following Environmental Stress. Nat. Commun. 8 (1), 1122. 10.1038/s41467-017-01195-y 29066820 PMC5654764

[B106] Young J. I. Züchner S. Wang G. (2015). Regulation of the Epigenome by Vitamin C. Annu. Rev. Nutr. 35, 545–564. 10.1146/annurev-nutr-071714-034228 25974700 PMC4506708

[B107] Yu H. Dai Z. (2019). SNNRice6mA: A Deep Learning Method for Predicting DNA N6-Methyladenine Sites in Rice Genome. Front. Genet. 10, 1071. 10.3389/fgene.2019.01071 31681441 PMC6797597

[B108] Zhang G. Diao S. Song Y. He C. Zhang J. (2022a). Genome-wide DNA N6-Adenine Methylation in Sea Buckthorn (Hippophae Rhamnoides L.) Fruit Development. Tree Physiol. 10.1093/treephys/tpab177 34986489

[B109] Zhang G. Huang H. Liu D. Cheng Y. Liu X. Zhang W. (2015). N6-methyladenine DNA Modification in Drosophila. Cell. 161 (4), 893–906. 10.1016/j.cell.2015.04.018 25936838

[B110] Zhang H. Lang Z. Zhu J.-K. (2018a). Dynamics and Function of DNA Methylation in Plants. Nat. Rev. Mol. Cell. Biol. 19 (8), 489–506. 10.1038/s41580-018-0016-z 29784956

[B111] Zhang L. Rong W. Ma J. Li H. Tang X. Xu S. (2022b). Comprehensive Analysis of DNA 5-Methylcytosine and N6-Adenine Methylation by Nanopore Sequencing in Hepatocellular Carcinoma. Front. Cell. Dev. Biol. 10, 827391. 10.3389/fcell.2022.827391 35321246 PMC8937020

[B112] Zhang M. Yang S. Nelakanti R. Zhao W. Liu G. Li Z. (2020). Mammalian ALKBH1 Serves as an N6-mA Demethylase of Unpairing DNA. Cell. Res. 30 (3), 197–210. 10.1038/s41422-019-0237-5 32051560 PMC7054317

[B113] Zhang Q. Liang Z. Cui X. Ji C. Li Y. Zhang P. (2018b). N6-Methyladenine DNA Methylation in Japonica and Indica Rice Genomes and its Association with Gene Expression, Plant Development, and Stress Responses. Mol. plant 11 (12), 1492–1508. 10.1016/j.molp.2018.11.005 30448535

[B114] Zhang Y. Liu Y. Xu J. Wang X. Peng X. Song J. (2021). Leveraging the Attention Mechanism to Improve the Identification of DNA N6-Methyladenine Sites. Briefings Bioinforma. 22, 351. 10.1093/bib/bbab351 PMC8575024 34459479

[B115] Zhou C. Wang C. Liu H. Zhou Q. Liu Q. Guo Y. (2018). Identification and Analysis of Adenine N6-Methylation Sites in the Rice Genome. Nat. plants 4 (8), 554–563. 10.1038/s41477-018-0214-x 30061746

[B116] Zhu S. Beaulaurier J. Deikus G. Wu T. P. Strahl M. Hao Z. (2018). Mapping and Characterizing N6-Methyladenine in Eukaryotic Genomes Using Single-Molecule Real-Time Sequencing. Genome Res. 28 (7), 1067–1078. 10.1101/gr.231068.117 29764913 PMC6028124

